# LINC01315 accelerates the growth and epithelial-mesenchymal transition of colorectal cancer cells via activating the Wnt/β-catenin signal

**DOI:** 10.1080/21655979.2022.2044275

**Published:** 2022-03-24

**Authors:** Yang Liu, Wen Li Zhou

**Affiliations:** Department of Gastroenterology, Guangrao County People’s Hospital, Dongying, Shandong, China

**Keywords:** CRC, LINC01315, tumorigenesis, EMT, wnt/β-catenin

## Abstract

The pathological roles of long non-coding RNAs (lncRNAs) in colorectal carcinoma (CRC) have been corroborated. To date, the pathological contributions of LINC01315 in the epithelial-mesenchymal transition (EMT) property of CRC are still ambiguous. By silencing LINC01315, we disclosed that LINC01315 promoted the growth, metastatic characteristics, and the EMT of CRC cells *in vitro*. Mechanistically, LINC01315 activated Wnt/β-catenin signaling. LINC01315 bound to the β-catenin promoter and activated its transcription. In rescue experiments, ectopic overexpression of β-catenin counteracted the inhibiting effector-triggered by LINC01315 deletion. In summary, this preliminary study brings new insights to the pathological significance of the LINC01315/Wnt/β-catenin signaling pathway in CRC.

## GRAPHICAL ABSTRACT

**Figure:**
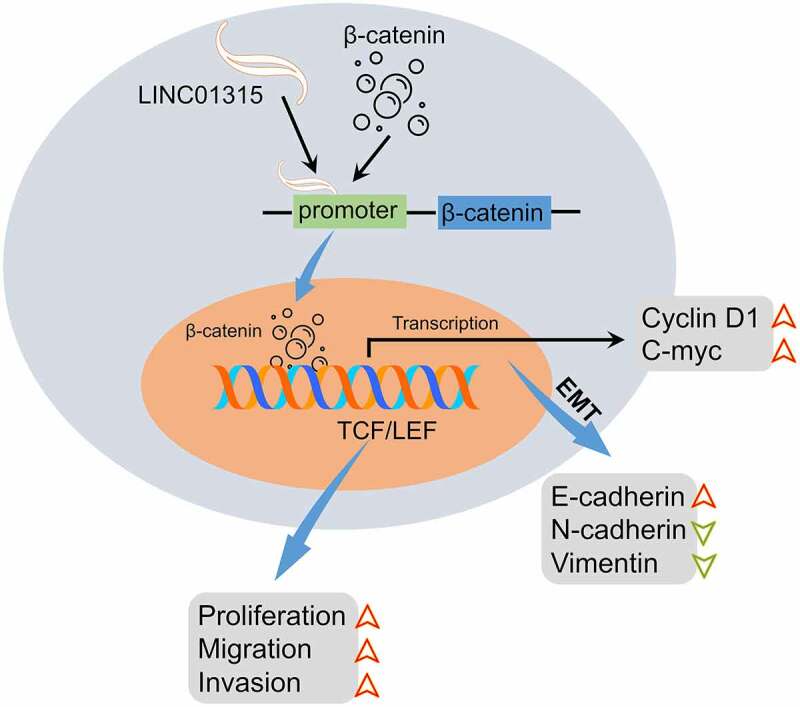

## Introduction

Colorectal carcinoma (CRC), one of the most frequent gastrointestinal malignancies, remains the primary trigger of malignancy-related mortality [1,2]. The major cause of CRC-caused mortality is distant metastasis, and approximately 60% of patients with CRC are expected to develop metastases [3]. The presence of metastasis confers a worse prognosis to the clinical outcome of CRC-affected patients [4]. An extensive body of studies has already focused on oncogenes and tumor suppressor genes that involve metastasis [5–7]. It is well-accepted that cancer metastasis is a highly complicated, multistep process and tightly linked with epithelial-to-mesenchymal transition (EMT) [8]. Focusing on the agents involved in the EMT of CRC cells might aid the comprehension of metastatic dissemination [9].

Anomalous activated Wnt-β-catenin classic signaling is essential in neoplastic cells metastasis and EMT program [10]. Specifically, activating Wnt/β-catenin triggers the expression of EMT activators, including Zinc Finger E-Box Binding Homeobox 1 (ZEB1), Twist Family BHLH Transcription Factor 1 (Twist1), Slug, and Snail to initiate EMT [11]. There is increasing research to develop Wnt/β-catenin signal inhibitors to suppress Wnt/β-catenin activated EMT [12,13]. As such, deciphering the molecular basis of the Wnt/β-catenin signaling aberrantly activation may improve the treatments regimens for CRC.

Accumulating evidence disclosed that long noncoding RNAs (lncRNA) are implicated in diverse pathological processes, including cancers [14–16]. The abnormal lncRNAs regulate transcription and posttranscriptional through various biological mechanisms in carcinogenesis [17,18]. Recently, Xue, et al. demonstrated that a high level of LINC01315 results in a worse prognosis in patients with breast carcinoma [19]. In papillary thyroid cancer, LINC01315 facilitates the metastatic capacity of cancerous cells *via* the miR-497-5p-sponging role [19]. In papillary thyroid cancer, LINC01315 boosts the metastatic capacity of cancerous cells *via* the miR-497-5p-sponging role [20]. Acts as a competitive endogenous RNA (ceRNA), LINC01315 debilitates the malignant properties of CRC *via* directly sponging miR-205-3p [21]. Dozens of lncRNAs, such as Beta-1,3-Galactosyltransferase 5 Antisense RNA 1 (B3GALT5-AS1), Cytoskeleton Regulator RNA (CYTOR), and LINC01287, have been established to contribute to the EMT and metastasis process of CRC [22–24]. Yet, the exact mechanism by which LINC01315 boosts CRC EMT remains enigmatic.

Herein, we reported that inhibition of LINC01315 dramatically retards CRC cells’ growth and EMT event. Mechanistically, LINC01315 regulates β-catenin transcription *via* direct interaction with its promoter. We collectively establish that LINC01315 acts as a pro-oncogenic factor in CRC by activating the canonical Wnt/β-catenin signal.

## Materials and methods

### CRC tissues

31 pairs of paraffin-embedded colorectal cancer tissues and matched paracancerous tissues were purchased from Xian Ailina Biotechnology Co., Ltd (Xian, China). The clinical characteristics are provided in Table 1. Informed consent from all participants was obtained. The study protocol was granted by the Ethical Committee from Guangrao County People’s Hospital (No. GR2019-11). The ethical approvement can be found in the Supplementary material.

**Table 1.** The clinicopathological parameters in patients with CRC

Variables	Cases (n)
**Age (years)**	
≤50	6
>50	25
**Gender**	
Female	20
Male	11
**Tumor stage**	
I–II	27
III	4
**Metastasis**	
Negative	3
Positive	29

### Cell lines

CRC cells (LOVO, HT29, HCT116, and SW480) and normal colorectal mucosa cell FHC were purchased from ATCC (Rockville, USA). Cells were cultured in Dulbecco’s modified Eagle’s medium (DMEM) medium (Invitrogen, Carlsbad, CA, USA) and 10% fetal bovine serum (FBS) (Invitrogen) supplemented with 1% penicillin-streptomycin (Invitrogen) at 37°C under 5% CO_2_.

### Cell transfection

An shRNA plasmid targeting LINC01315 (abbreviated as shLINC01315), and nonspecific shRNA (shNC) were synthesized by RiboBio (Guangzhou, China). SW480 and HCT116 cells were transfected with indicated plasmids using Lipofectamine 2000 reagents (Invitrogen) for 48 h. The full-length sequence of LINC01315 or β-catenin was cloned into pcDNA3.1 plasmid (Invitrogen) to produce pcDNA3.1-LINC01315 or pcDNA3.1-β-catenin, respectively. HCT116 and SW480 cells were seeded in six-well plates. When cell confluence reached 80%, cells were transfected with pcDNA3.1-LINC01315 or pcDNA3.1-β-catenin using the Lipofectamine 2000 reagents (Invitrogen). Cells were collected and subject to the following assays after 48 h post-transfection.

### Cell proliferation

HCT116 or SW480 cells (1 × 10^4^) were cultured into 96 well microplates. 10 µL of Cell Counting Kit-8 (CCK-8) solution was added to the plates at different time points (24, 48, 72, or 96 h). After incubation for 2 h at 37°C, a microplate reader was used to determine the absorbance values (450 nm). In the clonogenic test, cells (2 × 10^3^/well) were plated into six-well plates. After 14 days, the visible colonies were counted through an inverted microscope after cell colonies were stained with 0.1% crystal violet.

### Migration and invasion assay

HCT116 or SW480 cells (1 × 10^5^) were seeded in 6-well dishes and cultured overnight to reach 90% confluency, and an artificial was made using a 200 µL tip. After washing with PBS, cells were recorded at 0 h or 48 h. The migration rate (%) = (0 h wound width −48 h wound width)/0 h wound width ×100. In the invasion assay, 200 µL of cell suspension (2 × 10^4^) were plated in the Transwell top compartment (8-μm membrane; BD Biosciences) with an FBS-free DMEM medium. 600 µL of 10% FBS medium was added into the lower compartment. After 24 h, the invading cells across the membrane were fixed and stained with crystal violet. The number of invading cells was counted under an inverted microscope.

### Real-time quantitative PCR (qRT-PCR)

The total RNAs were extracted using TRIzol solution (Invitrogen). cDNA synthesis was implemented using a Primescipt RT reagent kit (TaKaRa, Dalian, China). qRT-PCR was performed using an SYBR Premix ExTaq Real‐Time PCR Kit (TaKaRa). GAPDH was used as the internal reference, and data were calculated using the 2^−ΔΔCT^ method. Specific primer sequences are shown as following: LINC01315 (forward) 5′-CTGCTGAGCGATGAAGTGGA-3′ and (reverse) 5′-CTACAGCTGGAGGGAAACCG-3′; β-catenin (forward) 5′-ATGGACGTGGGCGAACTTC-3′ and (reverse) 5′-TTTGTTTCCGACGCATCTTCT-3′; C-myc (forward) 5′-AGGGATCGCGCTGAGTATAA-3′ and (reverse) 5′-TGCCTCTCGCTGGAATTACT-3′; Cyclin D1 (forward) 5′-GCTGCGAAGTGGAAACCATC-3′ and (reverse) 5′-CCTCCTTCTGCACACATTTGAA-3′; GAPDH (forward) 5′-TGTGGGCATCAATGGATTTGG-3′ and (reverse) 5′-ACACCATGTATTCCGGGTCAAT-3′.

### Western blot

Total proteins were extracted using RIPA buffer (Beyotime, Nanjing, China). 35 μg of protein lysates were subjected to electrophoretic separation using 8% SDS-PAGE and transferred onto a PVDF membrane (Bio-Rad, CA, USA). The blots were incubated with β-catenin, N-cadherin, E-cadherin, Lamin B1, or GAPDH antibody (1:1000, Abcam, USA) overnight at 4°C and then incubated with HRP-labeled secondary antibody (1:10,000, Beyotime) for 2 h. The protein bands were visualized using an ECL kit (Beyotime).

### TOP/FOP flash reporter assay

HCT116 and SW480 cells were co-transfected with pcDNA3.1-LINC01315 and β-catenin-LEF/TCF-sensitive (TOP) or LEF/TCF insensitive (FOP) reporter vector (Addgene) using Lipofectamine 2000 reagents (Invitrogen). A constitutively active Renilla luciferase construct (Promega) was transfected for normalization of transfection efficiency. 48 h post-transfection, a Dual-Luciferase Reporter System (Promega) was applied to assess the luciferase reporter activity.

### Luciferase reporter assay

Fragments of the β-catenin promoter (−2000 bp to +44 bp) were cloned into pGL3-Basic Vector (Promega). HCT116 or SW480 cells were plated into 24-well plates and transfected with the plasmids, concomitantly with pcDNA3.1-LINC01315 and a pRL-TK Renilla luciferase plasmid (Promega). 48 h after transfection, the luciferase activity was measured.

### RNA immunoprecipitation (RIP)

RIP assay was carried out using a Magna RIPTM RNA-Binding Protein Immunoprecipitation Kit (Millipore). Cell lysates were incubated with β-catenin of IgG antibody. Co-precipitated RNAs were enriched by Protein A/G beads. Finally, the coprecipitated RNAs were subjected to qRT-PCR assay.

### RNA pull-down

LINC01315 sequence was labeled in Biotin, and RNA pull-down was performed using a Pierce Magnetic RNA-Protein Pull-Down Kit (ThermoFisher, MA, USA). Cell lysates were incubated with the biotin-labeled LINC01315 for 4 h at 4°C and treated with magnetic beads for 30 min. After being washed three times, the beads were boiled and precipitated proteins were detected using immunoblotting.

### Statistical analysis

Statistical analysis was done using GraphPad Prism 7.0 (GraphPad Software, La Jolla, CA, USA). Data are shown as Mean ± SD. Kaplan‐Meier curve followed by the log‐rank test was applied to evaluate the prognosis of patients. One-way ANOVA tests or two-tailed Student’s t-test was used for statistical analysis, where indicated. *P* value<0.05 is considered significant.

## Results

In this study, we focused on the pro-cancer characteristics of LINC01315 in CRC. LINC01315 promotes the growth, metastatic properties, and the EMT of CRC cells *in vitro*. Mechanistically, LINC01315 binds to the β-catenin promoter and activates the Wnt/β-catenin signaling.

### LINC01315 is overexpressed in CRC

**Figure 1.**
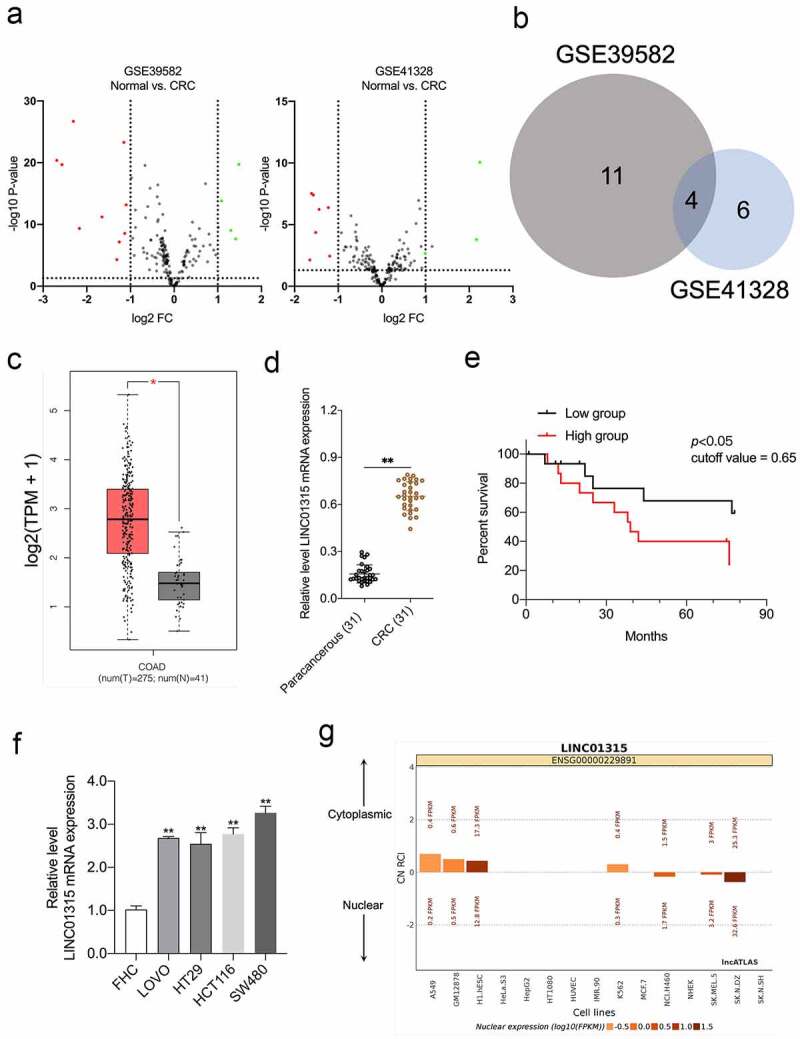
LINC01315 is upregulated in CRC. a. Volcano maps of differentially expressed lincRNAs in GSE39582 and GSE41328. Red spots represent upregulated lincRNAs; green spots represent downregulated lincRNAs. b. Overlap of the dysregulated lincRNAs among GSE39582 and GSE41328. c. The expression of LINC01315 was analyzed using TCGA CRC dataset. d. LINC01315 expression level was significantly higher in CRC tissues than paracancerous tissues. **P < 0.01 compared with paracancerous. e. The effect of LINC01315 expression on clinical prognosis was determined by Kaplan-Meier survival analysis. The median value (0.65) was the cutoff threshold of Kaplan-Meier survival analysis. f. Expression level of LINC01315 was significantly higher in CRC cell lines than the human colon immortalized cell line, FHC. **P < 0.01 compared with FHC. g. The subcellular localization of LINC01315 predicted on lncATLAS website (https://lncatlas.crg.eu/).

First, we assessed the aberrant lncRNAs expressions using the GEO database (GSE39582 and GSE41328) based on the filter conditions of *P* < 0.05 and |fold change| > 1 (Figure 1(a)) [25,26]. Venn diagram showing the intersection of the differential lncRNAs in the two GEO datasets (Figure 1(b)). The pathological roles of LINC00152, LINC00675, and LINC01207 in CRC have been delineated previously. Nonetheless, the pro-oncogenic action of LINC01315, especially in CRC EMT, has not been fully addressed [27–30]. In the public The Cancer Genome Atlas Program (TCGA) dataset, in contrast to normal, high LINC01315 was found in CRC (Figure 1(c)). We evaluated the expressions of LINC01315 in 31 pairs of CRC specimens and paracancerous tissues. LINC01315 was profoundly elevated in CRC compared with paracancerous tissues (Figure 1(d)). Notably, Kaplan-Meier survival analysis revealed that patients with high LINC01315 expression levels had poorer overall survival than patients with low LINC01315 expression levels (Figure 1(e)). Comparing the normal colorectal mucosa cell FHC, CRC cells (SW480, LOVO, HCT116, and HT29) exhibited higher LINC01315 expression (Figure 1(f)). Finally, LINC01315 is mainly located in the cytoplasm, as revealed by the lncATLAS website (https://lncatlas.crg.eu) (Figure 1(g)). Collectively, the above results implied that LINC01315 might act as a predictor of prognosis for CRC.

### Deletion of LINC01315 restrains CRC cell growth and mobility

**Figure 2.**
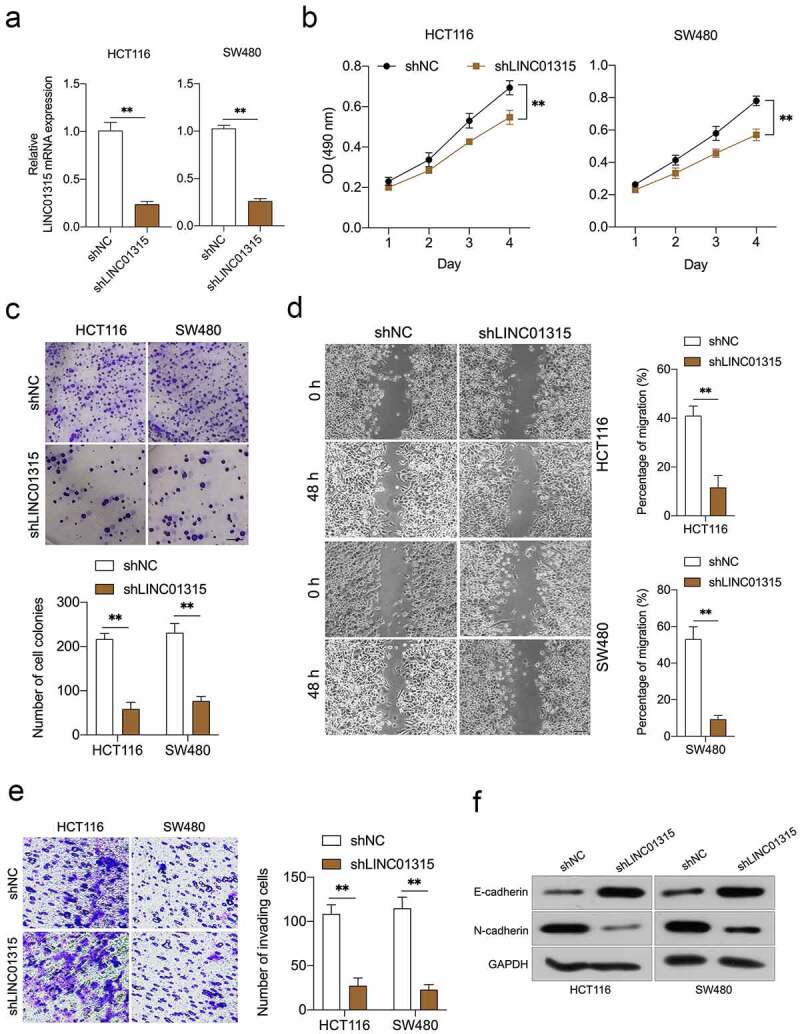
Deletion of LINC01315 inhibits CRC cells growth, invasion, and EMT. a. LINC01315 knockdown by shRNA (shLINC01315) was confirmed using qRT-PCR. b. CCK-8 assay showing proliferation of HCT116 and SW480 cancer cells following LINC01315 knockdown. c. Colony formation assays were performed to determine the proliferation of shLINC01315-transfected or shNC- transfected CRC cells. d. Wound-healing assay of LINC01315 knockdown HCT116 and SW480 cells. e. Transwell invasion assay analyzed the effect of LINC01315 knockdown on CRC cells invasion. f. Western blot assay was performed to test the expression of EMT markers (E-cadherin and N-cadherin) in the LINC01315 knockdown cells. **P < 0.05 compared with shNC.

To figure out the biological role of LINC01315 in CRC progression, shLINC01315 was applied to knock down the endogenous LINC01315 expression in HCT116 and SW480 cells (Figure 2(a)). CCK-8 assays showed that LINC01315 silencing caused dramatic growth inhibition in CRC cells (Figure 2(b)). A similar suppressive action was observed in the clonal formation ability of CRC cells up LINC01315 knockdown (Figure 2(c)). In addition, wound healing and transwell assays implied that attenuation of LINC01315 expression impaired CRC cells’ migration (Figure 2(d)) and invasive abilities (Figure 2(e)). Besides, epithelial marker E-cadherin expression was raised by LINC01315 deficiency, while the protein expression of the mesenchymal marker, N-cadherin, was lessened (Figure 2(f)). All these data validated that attenuation of LINC01315 expression prohibits CRC EMT and invasion.

### LINC01315 activates Wnt/β-catenin in CRC

**Figure 3.**
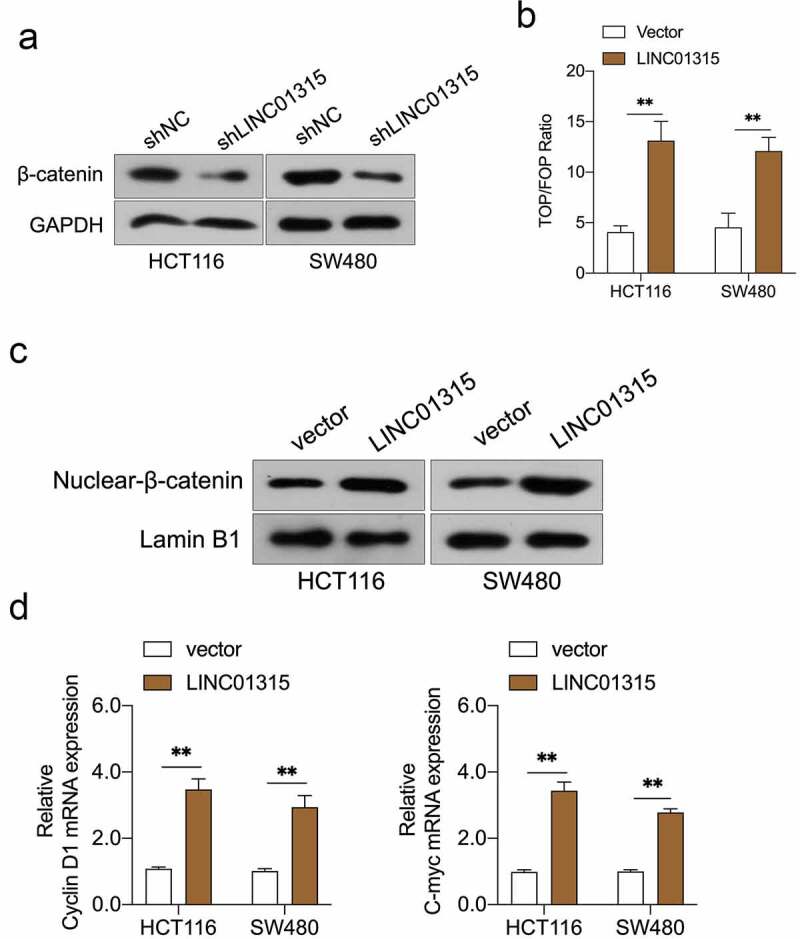
LINC01315 activates the Wnt/β-catenin pathway. a. LINC01315 knockdown results in a reduction of β-catenin expression. b. TOP/FOP luciferase reporter assays were performed to detect the β-catenin activity. c. Altered nuclear translocation of β-catenin in response to LINC01315 overexpression. d. Upregulation of LINC01315 raised the RNA levels on Wnt/β-catenin direct-target genes. **P < 0.05 compared with vector.

Wnt/β-catenin signal is implicated in cancer metastasis and EMT [31]. The Western blot results indicated that the silencing of LINC01315 substantially reduced the protein expression of β-catenin (Figure 3(a)). To probe whether LINC01315 activated Wnt/β-catenin, the TOP/FOP flash luciferase reporter assay in CRC cells was conducted. The result indicated that the introduction of LINC01315 activated the Wnt/β‐catenin pathway (Figure 3(b)). Next, we assayed the nuclear/cytoplasmic localization of β-catenin in LINC01315 overexpressing CRC cells. As presented in Figure 3(c), force expression of LINC01315 increased β-catenin nucleus ectopic expression. Similarly, the mRNA levels of Wnt/β-catenin target genes, Cyclin D1 and C-myc, were raised in CRC cells upon LINC01315 overexpression (Figure 3(d)).

### LINC01315 interacts β-catenin in CRC cells

**Figure 4.**
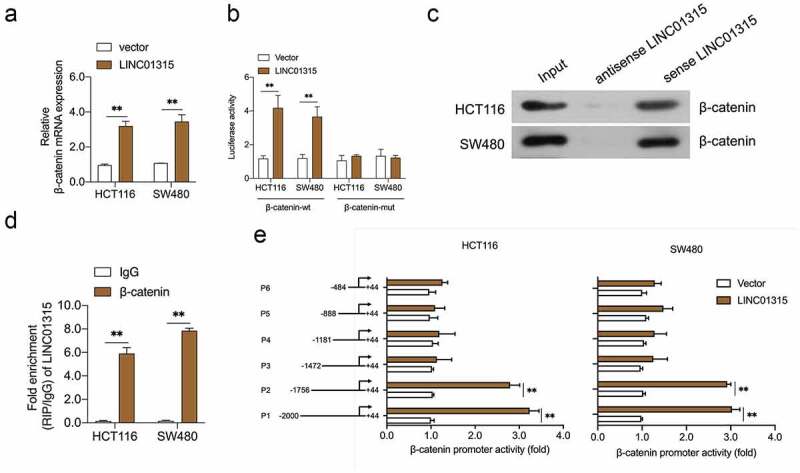
LINC01315 binds to the β-catenin promoter. a. pcDNA3.1-LINC01315 transfection increased β-catenin mRNA level. b. Relative luciferase activities of the β-catenin reporter in LINC01315 overexpressing CRC cells. c. The targeting relation of LINC01315 and β-catenin was confirmed by RNA pull-down assay. d. RIP was performed to measure the binding abilities of LINC01315 and β-catenin. e. The activity of the β-catenin promoter was measured by luciferase assay. **P < 0.05 compared with vector.

LncRNAs have been reported to regulate genes expression at the transcriptional level, the impact of LINC01315 on β-catenin was further examined. As shown in Figure 4(a), upregulation of LINC01315 resulted in an increased expression of β-catenin at the mRNA level. Subsequently, the luciferase reporter assay proved the activating effect of LINC01315 in the β-catenin promoter (Figure 4(b)). To seek the mechanism by which LINC01315 regulates β-catenin transcription, the pull-down assay was executed, and we discovered that LINC01315 precipitated the endogenous β-catenin in CRC cells (Figure 4(c)). Similarly, the RIP assay suggested that the endogenous LINC01315 was enriched by β-catenin antibody (Figure 4(d)). Finally, serial truncated promoter promoters were constructed. As indicated in Figure 4(e), the luciferase activities of P1 (−2000 to +44) and P2 (−1756 to +44) promoters were increased upon LINC01315 overexpression. The luciferase activities of P3 (−1472 to +44), P4 (−1188 to +44), P5 (−888 to +44), and P6 (−484 to +44) were not upregulated by LINC01315. These data implied that the sequences (−1756 and −1473) of the β-catenin promoter contain a LINC01315 binding site.

### *LINC01315 promotes CRC invasion and EMT* via *activating wnt/β-catenin*

**Figure 5.**
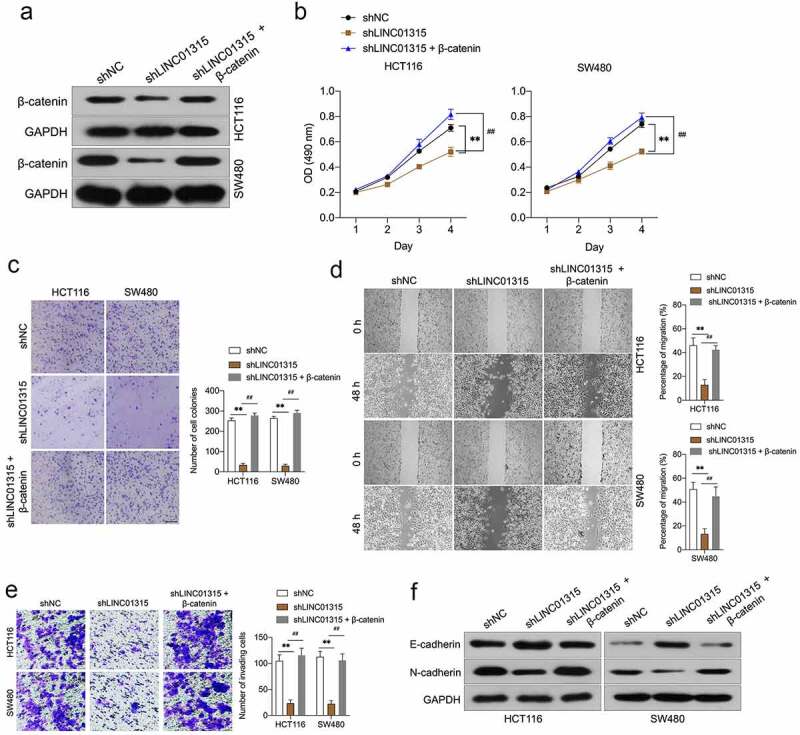
Overexpression of β-catenin reverses the effects of LINC01315 on CRC cells EMT. a. HCT116 and SW480 cells were co-transfected with shLINC01315 and pcDNA3.1-β-catenin. The expression of β-catenin was determined by Western blot. b. CCK-8 assay showing proliferation of HCT116 and SW480 cells. **C**. Colony formation assays were performed to determine the proliferation of shLINC01315 and pcDNA3.1-β-catenin co-transfected CRC cells. d. Wound-healing assay of HCT116 and SW480 cells. e. Transwell invasion assay analyzed CRC cells invasion. f. Western blot assay was performed to test the expression of EMT markers (E-cadherin and N-cadherin) in HCT116 and SW480 cells. **P < 0.05 compared with shNC, ^##^*P* < 0.05 compared with shLINC01315.

To confirm LINC01315-induced augment of CRC cells invasion and EMT was dependent on β-catenin. LINC01315-silenced CRC cells were transfected with pcDNA3.1-β-catenin plasmid to restore the expression of β-catenin (Figure 5(a)). In Figure 5(b-e)), the proliferation, clonogenic ability, migration, and invasiveness suppressed by LINC01315 inhibition were rescued by pcDNA3.1-β-catenin in CRC cells. Inhibition of LINC01315 decreased N-cadherin and increased the expression of E-cadherin. However, this tendency was countered by β-catenin overexpression (Figure 5(f)). These above observations implied that LINC01315 exerts pro-oncogenic functions is dependent on Wnt/β-catenin signaling in CRC.

## Discussion

Emerging studies have focused on the cancer-specific lncRNAs and illuminated their imperative roles in cancer progression [14,15]. In this work, we elucidated the pathomechanism of LINC01315 in CRC EMT. LINC01315 is abnormally high in CRC and acts as an unfavorable prognostic marker for CRC. LINC01315 silencing suppresses the migration and invasion of CRC cells and diminishes the EMT process.

Ectopic lncRNAs expressions have been implicated in cancer metastasis through the downstream effector molecules deregulation [32]. LINC01315 elevates the expression of discs large homolog 3 (DLG3) *via* sponging miR-211 and then delays oral squamous cell carcinoma (OSCC) progression [33]. Patients with a high LINC01315 expression level are associated with worse clinical outcomes, and LINC01315 might be an independent prognostic predictor in breast cancer [19]. As a ceRNA, inhibition of LINC01315 attenuates the aggressive phenotypes of CRC through sponging the miR-205-3p [21]. In papillary thyroid cancer (PTC), LINC01315 sponges miR-497-5p and fortifies the metastatic behaviors of PTC cells [20]. The way of lncRNA exerts the function mainly depends on its subcellular localization. Except for the ceRNA network of lncRNAs-miRNAs, lncRNAs serve as oncogenic regulators through alternative mechanisms, including transcriptional, posttranscriptional regulation, and chromatin modification [34,35].

Although early studies show LINC01315 participates in the regulation of CRC cells’ mobility and invasion, the potential involvement of LINC01315 in CRC EMT has never been proposed before [21]. Deregulation of the Wnt/β-catenin signaling axis plays a significant role in the EMT and is required for colorectal tumor metastasis [1]. Herein, we disclosed that LINC01315 positively regulates EMT and activates the Wnt/β-catenin signaling. The expressions of mesenchymal markers and the downstream targets (C-myc and Cyclin D1) of the Wnt/β-catenin signal are raised in CRC cells upon LINC01315 overexpression. Subsequently, we found that LINC01315 inhibition substantially declined the level of β-catenin at both protein and mRNA levels, indicating that LINC01315 transcriptionally regulates β-catenin expression.

Mechanistically, LINC01315 directly binds to the promoter site of β-catenin, as demonstrated by the luciferase reporter gene assay. Further experiment with truncated constructs of β-catenin promoter indicates that LINC01315 binding site exists the sequence between the nucleotides −1756 and −1473 in the β-catenin promoter. Notably, our results showed that LINC01315 knockdown repressed CRC EMT and invasion, while β-catenin overexpression partially reversed the inhibition effects caused by LINC01315 knockdown.

There are some deficiencies in the presented study. The results obtained are solely based on *in vitro* experiments. For the most part, *in vitro* experiments will still need to be followed up with in *vivo* experiments. In the follow-up work, we will proceed with a xenograft tumor model to explore the anti-metastasis of LINC01315 *in vivo*. In fact, the degree of β-catenin phosphorylation impacts the stability of β-catenin and directly alters the functions of the Wnt/β-catenin signal. Moreover, an increased Glycogen Synthase Kinase-3β (GSK-3β) stability diminishes the Wnt/β-catenin pathway *via* phosphorylating β-catenin [12]. Further analysis is required to determine the impact of LINC01315 on GSK-3β and β-catenin phosphorylation.

## Conclusion

Collectively, we delineated that LINC01315 was exceptionally high in CRC and increased expression of LINC01315 predicted a poor prognosis in human CRC. Loss-of-function experiments demonstrated that LINC01315 fortified CRC cells migration, invasion, and EMT *in vitro*. Further analysis indicated that LINC01315 exerts pro-oncogenic functions by promoting the transcriptional activation of β-catenin. This study yields new insights into the role of LINC01315/Wnt/β-catenin in CRC EMT.

## Data Availability

The datasets used or/and analyzed during the current study are available from the corresponding author on reasonable request.
